# Trends in the Management of Acute Pyogenic Flexor Tenosynovitis of the Hand: A Comprehensive Analysis

**DOI:** 10.7759/cureus.109637

**Published:** 2026-05-25

**Authors:** Michael Anderson, Taylor J Manes, Davis Hedbany, Sylvester Okoro, Nithin Gupta, Morgan Turnow, Stephen Berling

**Affiliations:** 1 Department of Orthopedic Surgery, OhioHealth, Columbus, USA; 2 Department of Orthopedics, OhioHealth Doctors Hospital, Columbus, USA; 3 Department of Orthopedic Surgery, SUNY Upstate Medical University, Syracuse, USA; 4 Department of Orthopedic Surgery, Morehouse School of Medicine, Atlanta, USA; 5 Department of Orthopedic Surgery, Campbell University School of Osteopathic Medicine, Buies Creek, USA

**Keywords:** amputation, apft, finger infection, hand surgery, kanavel signs, pyogenic flexor tenosynovitis, risk factors

## Abstract

Background

Acute pyogenic flexor tenosynovitis (APFT) is typically managed operatively, but there is growing interest in nonoperative treatments using intravenous (IV) antibiotics to reduce surgical complications. Timely intervention is essential, as delayed treatment increases the risk of systemic, life-threatening infections. This study aimed to characterize national trends in the operative management of APFT and to compare mortality and amputation outcomes between operatively and nonoperatively managed patients.

Methods

A retrospective analysis was conducted using data from the TriNetX United States Collaborative Network from 2015 to 2023. Patients were identified using ICD-10 and CPT codes and categorized into operative and nonoperative cohorts, further stratified by body mass index (BMI) (20-29 and 30-39). Primary outcomes included the incidence and prevalence of surgical interventions, while secondary outcomes assessed mortality, amputation rates, and the risk of surgery and reoperation based on BMI. Propensity score matching was performed to control for confounders.

Results

The study included 2,200 operative and 4,099 nonoperative patients. Risk factors such as nicotine dependence, tobacco use, and diabetes increased the likelihood of surgery. From 2015 to 2023, the incidence of tendon sheath drainage rose from 0.59% to 13.29%, with surgical prevalence increasing to 35.38%. Operative management - likely reflecting more severe infection at presentation - was associated with higher five-year mortality (OR 1.284) and amputation (OR 2.400). Surgery rates were significantly higher in the BMI 30-39 group.

Conclusion

APFT poses a serious risk, with increased surgery rates linked to certain risk factors. Educating patients on modifiable risks is critical for reducing APFT incidence.

## Introduction

The flexor tendon sheaths of the hand encompass a vital anatomical feature involved in acute pyogenic flexor tenosynovitis (APFT), allowing for the smooth movement of tendons during finger flexion. Anatomically, these sheaths extend from the distal palm to the distal phalanges, enveloping the flexor tendons. APFT occurs when these sheaths become infected, often leading to significant swelling and pain [1]. This morbid condition necessitates prompt and effective management to prevent adverse outcomes such as tendon damage and functional impairment. Predominantly caused by *Staphylococcus aureus*, this malady manifests through intense pain, swelling, and limited finger movement, typically following direct inoculation injuries [1]. With a significant burden on hand function, contemporary management strategies have evolved from strictly surgical interventions to conservative approaches that prioritize early diagnosis and tailored antibiotic therapies [2,3].

APFT classically presents with four physical exam characteristics, also known as the Kanavel signs, which include fusiform swelling of the finger, pain along the flexor tendon sheath, a finger held in slight flexion, and pain on passive extension of the finger, which has been shown to be the most sensitive physical exam finding [4]. These clinical manifestations are crucial for early diagnosis and prompt treatment to prevent further complications.

APFT predominantly affects populations with certain predisposing factors that compromise the integrity of the skin and immune defenses, including intravenous (IV) drug users, people with diabetes, and individuals with compromised hygiene [5]. Additionally, smokers are at a higher risk due to the peripheral vascular and immune system effects of tobacco use [3,5].

Demographically, the incidence of APFT varies across regions, ages, genders, races, and ethnicities. For instance, regional data may reflect higher incidence rates in areas with large at-risk populations. Age also plays a role, with middle-aged adults frequently experiencing higher occurrence rates, possibly linked to increased engagement in manual labor or a higher prevalence of diabetes and smoking in this age group [6]. Males are slightly more affected than females, likely due to occupational hazards or lifestyle factors. Racial and ethnic disparities in APFT occurrence could also reflect underlying socioeconomic and healthcare accessibility issues, influencing exposure risks and treatment outcomes [6,7].

There has been increasing interest in exploring select nonoperative management strategies for early APFT, primarily involving IV antibiotics alone [8]. This approach has been considered only in carefully chosen patients diagnosed early, without abscess formation or systemic illness. Studies suggest that, in these limited circumstances, aggressive antibiotic therapy and close monitoring may yield outcomes comparable to surgery [9,10].

Additionally, the timing of intervention after infection plays a critical role in patient outcomes. With increasing delay before the initiation of treatment, there is a greater risk of adverse outcomes, including systemic infections that can be life-threatening [11]. Therefore, timely medical intervention is crucial for improving survival rates and preventing the spread of infection to other parts of the body.

The primary objective of this study was to characterize national trends in the incidence and prevalence of operative intervention for APFT from 2015 to 2023. Secondary objectives were to compare mortality, amputation, and body mass index (BMI)-stratified surgical risk between operatively and nonoperatively managed patients.

This article was previously presented as a meeting poster at the 2026 AAOS Meeting on March 4, 2026.

## Materials and methods

Study design and data source

This retrospective study was performed using data from January 2015 to December 2023 obtained from the TriNetX United States Collaborative Network, a comprehensive database that includes electronic medical records from approximately 113 million patients across 64 healthcare organizations. The TriNetX platform provides access to diagnoses, procedures, medications, laboratory values, and genomic information through real-time, de-identified data. This study, queried on May 8, 2024, did not require IRB approval, as the data collected were de-identified and compliant with the Health Insurance Portability and Accountability Act (HIPAA).

Study population

The APFT study population was defined using the International Classification of Diseases, 10th edition (ICD-10), diagnosis codes for abscess of the tendon sheath (M65.041, M65.042, and M65.049) and infective tenosynovitis (M65.141, M65.142, and M65.149). Eligible patients were over the age of 18 years and diagnosed with APFT between 2015 and 2023. Surgical intervention was identified using the Current Procedural Terminology (CPT) code 26020 for tendon sheath drainage. Patients were categorized into two cohorts based on surgical status and further stratified by BMI categories: 20-29 (Z68.2) and 30-39 (Z68.3).

Study outcomes and statistical analyses

The primary outcomes included the incidence and prevalence of surgical interventions in the treatment of APFT from 2015 to 2023, expressed as the annual proportion of diagnosed APFT patients undergoing tendon sheath drainage. Baseline demographic and risk factor comparisons between surgical and non-surgical cohorts included regional data, age, gender, race, ethnicity, BMI, nicotine dependence, tobacco use, and diabetes.

A secondary analysis was performed to measure mortality rates at one, three, and five years post-diagnosis; amputation rates between the operative and non-operative cohorts; and the risk of initial surgery and re-operation across the two BMI stratifications. Amputation outcomes were analyzed for patients undergoing the following procedures: direct closure (26951), local advancement flaps (26952), ray amputation (26910), or any combination of these techniques.

Categorical outcomes were analyzed using z-tests for proportions, and continuous outcomes were analyzed using two-sided independent-sample t-tests. All data management and statistical analyses were conducted within the TriNetX analytics platform, and statistical significance was defined as a two-sided p-value < 0.05.

To control for potential confounders, propensity score matching was implemented, with patients matched by age, gender, race, and ethnicity to ensure that comparisons between cohorts were as fair and unbiased as possible, given the observational nature of the data. Matching was performed within the TriNetX platform using 1:1 greedy nearest-neighbor matching on logistic-regression-derived propensity scores with a caliper of 0.10 pooled standard deviations. Consistent with administrative-database methodology, the absence of a coded record was treated as the absence of that characteristic rather than being imputed. The operative cohort comprised patients with CPT 26020 at any point during the study window, whereas the nonoperative cohort comprised diagnosed patients without that code.

The total cohort included 6,299 patients diagnosed with APFT. After stratification by surgical status, 2,200 patients were in the operative group and 4,099 were in the non-operative group. Following propensity-score matching, the cohorts for mortality outcome analysis each comprised 2,198 patients. For the BMI-related analyses, the groups initially included 790 patients with a BMI of 20-29 and 969 patients with a BMI of 30-39. After propensity-score matching, 733 patients remained.

## Results

Characteristics

A total of 2,200 patients were included in the operative group, while 4,099 were categorized in the non-operative group. Operative patients were predominantly from the Southern United States (46%), while non-operative patients were mainly from the Northeastern United States (32%) (Table 1). Demographically, patients in the operative group were significantly younger, more likely to be male, and predominantly non-Hispanic or Latino compared to the non-operative group (Table 2). Additionally, a higher proportion of the operative group was identified as White patients. Several risk factors were associated with a higher likelihood of undergoing surgery, including nicotine dependence (from both cigarettes and unspecified sources), tobacco use, and both type 1 and type 2 diabetes mellitus. It is important to note that there was no significant difference in BMI as a risk factor for undergoing the operation between the two groups.

**Table 1:** Regional characteristics of patients stratified by their operative status.

Region	Operative group (n, %)	Non-operative group (n, %)
Northeast	554 (25.2%)	1,324 (32.3%)
Midwest	294 (13.4%)	443 (10.8%)
South	1,019 (46.3%)	1,272 (31.0%)
West	329 (14.9%)	728 (17.8%)
Unknown	4 (0.2%)	332 (8.1%)

**Table 2:** Comparative demographic characteristics of patients in the operative and non-operative groups. Continuous variables were compared using independent-samples t-tests, and categorical variables were compared using two-proportion z-tests. SD, standard deviation; BMI, body mass index

Baseline characteristics comparison				
	Operative group (N = 2,200 patients), n (%)	Non-operative group (N = 4,099), n (%)	Test statistic	p-value
Current age	52.1 ± 16.1 years	55.2 ± 16.8 years	-7.1749	0.0001
Male	1,323 (60.1%)	2,298 (56.1%)	3.118	0.0018
Female	795 (36.1%)	1,557 (38.0%)	-1.446	0.1482
Unknown gender	82 (3.7%)	244 (6.0%)	-3.8009	0.0001
Not Hispanic or Latino	1,690 (76.8%)	2,773 (67.7%)	7.6329	0.0001
Hispanic or Latino	230 (10.5%)	307 (7.5%)	4.0172	0.0001
Unknown ethnicity	280 (12.7%)	1,019 (24.9%)	-11.3461	0.0001
White patients	1,567 (71.2%)	2,777 (67.7%)	2.8453	0.0044
Black or African American patients	268 (12.2%)	514 (12.5%)	-0.4106	0.6814
Unknown race	213 (9.7%)	519 (12.7%)	-3.5181	0.0004
Other race	82 (3.7%)	138 (3.4%)	0.7432	0.4574
Asian	38 (1.7%)	93 (2.3%)	-1.4359	0.151
Native Hawaiian or Other Pacific Islander	16 (0.7%)	41 (1.0%)	-1.0907	0.2754
American Indian or Alaska native	16 (0.7%)	17 (0.4%)	1.6381	0.1014
Body mass index (BMI) 19.9 or less, adult	82 (3.7%)	136 (3.3%)	0.8474	0.3968
Body mass index (BMI) 20-29, adult	263 (12.0%)	552 (13.5%)	-1.7047	0.0882
Body mass index (BMI) 30-39, adult	343 (15.6%)	650 (15.9%)	-0.2768	0.7819
Body mass index (BMI) 40 or greater, adult	163 (7.4%)	294 (7.2%)	0.3451	0.73
Nicotine dependence, cigarettes	747 (34.0%)	1,287 (31.4%)	2.0688	0.0386
Nicotine dependence, unspecified	652 (29.6%)	1,099 (26.8%)	2.3858	0.017
Tobacco use	459 (20.9%)	741 (18.1%)	2.6844	0.0073
Type 2 diabetes mellitus	722 (32.8%)	1,232 (30.1%)	2.2592	0.0239
Type 1 diabetes mellitus	190 (8.6%)	294 (7.2%)	2.0797	0.0376

Incidence and prevalence

**Figure 1:**
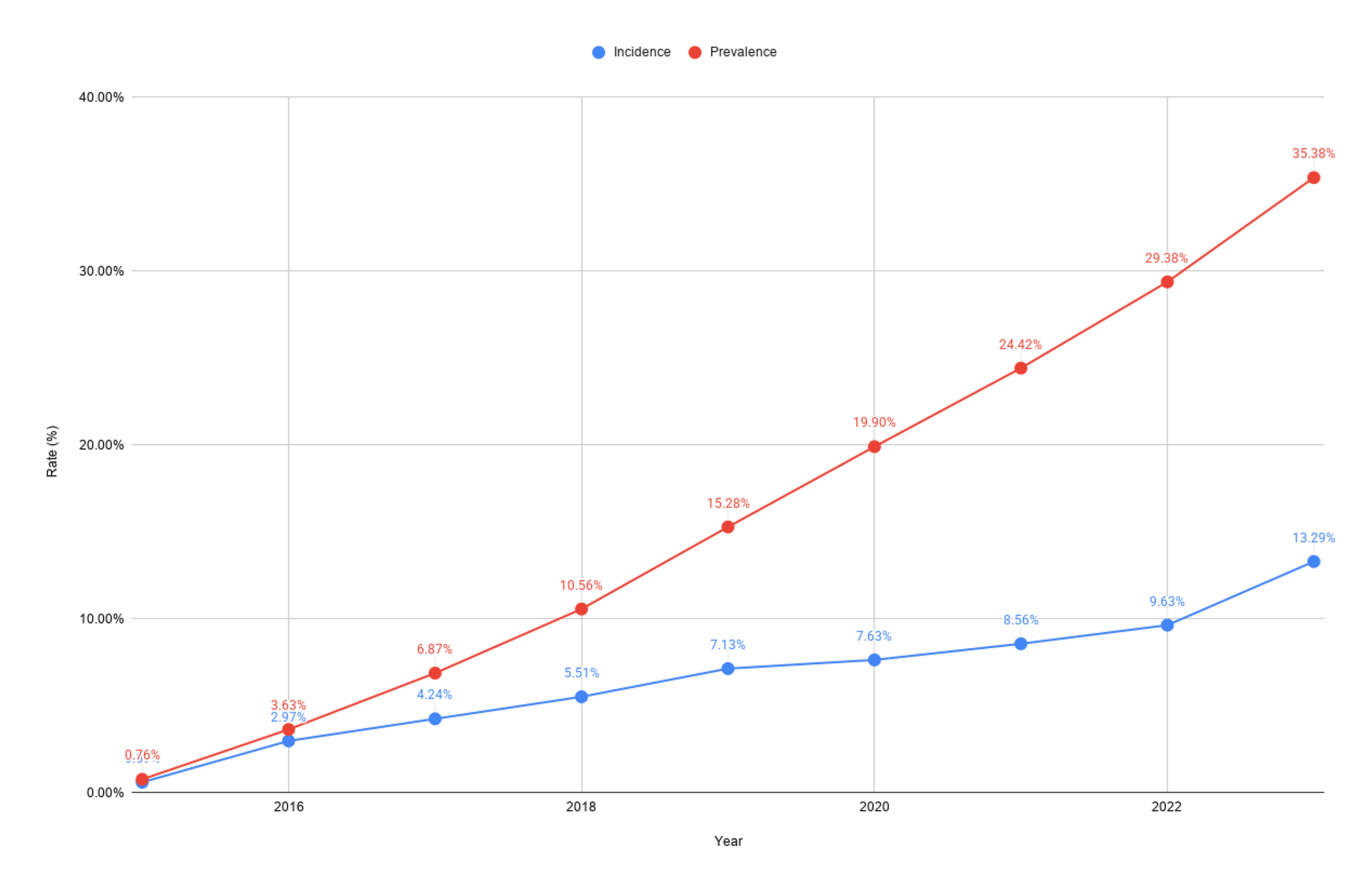
Overall incidence and prevalence trends of all APFT surgeries from 2015 to 2023. APFT, acute pyogenic flexor tenosynovitis

The analysis of the incidence and prevalence of tendon sheath drainage for treating APFT demonstrates a progressive increase in surgical interventions. The incidence of tendon sheath drainage rose from 21 (0.59%) in 2015 to 345 (13.29%) in 2023 (Table 3 and Figure 1). Prevalence increased from 27 (0.76%) in 2015 to 1,232 (35.38%) in 2023.

**Table 3:** Overall incidence and prevalence trends of all APFT surgeries from 2015 to 2023. APFT, acute pyogenic flexor tenosynovitis

Year	2015	2016	2017	2018	2019	2020	2021	2022	2023
Incidence (no. of cases)	21 (0.59%)	117 (2.97%)	183 (4.24%)	244 (5.51%)	314 (7.13%)	314 (7.63%)	328 (8.56%)	321 (9.63%)	345 (13.29%)
Prevalence (no. of cases)	27 (0.76%)	144 (3.63%)	305 (6.87%)	494 (10.56%)	738 (15.28%)	945 (19.9%)	1133 (24.42%)	1254 (29.38%)	1232 (35.38%)

Mortality rate

This study evaluated the one-year, three-year, and five-year mortality rates between operative and non-operative groups before and after propensity score matching. In the unmatched cohort, the one-year and three-year mortality odds ratios (ORs) did not reach statistical significance. Specifically, the one-year OR was 1.298 (95% CI, 0.938 to 1.798), and the three-year OR was 1.252 (95% CI, 0.987 to 1.589). However, at the five-year mark, the mortality OR of 1.318 (95% CI, 1.065 to 1.631) indicated an increased risk of mortality in the operative group, which was statistically significant.

After propensity score matching, the results were similar in terms of statistical significance. The one-year and three-year mortality rates remained non-significant with ORs of 1.350 (95% CI, 0.921 to 1.980) and 1.281 (95% CI, 0.971 to 1.690), respectively (Table 4). At the five-year interval, the mortality rate demonstrated a statistically significant increased risk with an OR of 1.284 (95% CI, 1.004 to 1.643).

**Table 4:** Matched 1, 3, and 5-year mortality rates of patients within the operative and non-operative cohorts. ORs were derived from logistic regression analyses. OR, odds ratio; CI, confidence interval

Cohort	No. of patients	1-year mortality (no. of patients)	OR (CI)	3-year mortality (no. of patients)	OR (CI)	5-year mortality (no. of patients)	OR (CI)
Operative Group	2,198	63	1.350 (0.921, 1.980)	119	1.281 (0.971, 1.690)	153	1.284 (1.004, 1.643)
Non-operative Group	2,198	47		94		121	

Amputation

We evaluated the likelihood of finger or thumb amputation in patients with APFT between the operative and non-operative groups. In the unmatched cohort, the operative group was significantly more likely to undergo amputation compared to the non-operative group. Specifically, the OR for any procedure was 2.495 (95% CI, 1.994 to 3.120), direct closure was 2.498 (95% CI, 1.907 to 3.272), local advancement flaps were 2.661 (95% CI, 1.476 to 4.796), and ray amputation was 2.409 (95% CI, 1.664 to 3.489). After propensity score matching, operative patients continued to show a significantly higher likelihood of undergoing amputation procedures compared to non-operative patients (Table 5). Specifically, the OR for any procedure was 2.400 (95% CI, 1.836 to 3.138), direct closure was 2.559 (95% CI, 1.838 to 3.562), local advancement flaps were 2.721 (95% CI, 1.314 to 5.634), and ray amputation was 2.130 (95% CI, 1.383 to 3.280).

**Table 5:** Matched likelihood for patients to undergo amputation within the operative and non-operative groups. ORs were derived from logistic regression analyses. 26951: direct closure surgical amputation; 26952: local advancement flaps with surgical amputation; 26910: ray amputation OR, odds ratio; CI, confidence interval

Cohort	No. of patients	26951, 26952, OR 26910	OR (CI)	26951 only	OR (CI)	26952 only	OR (CI)	26910 only	OR (CI)
Operative Group	2,198	185	2.400 (1.836, 3.138)	126	2.559 (1.838, 3.562)	27	2.721 (1.314, 5.634)	65	2.130 (1.383, 3.280)
Non-operative Group	2,198	81		51		10		31	

BMI outcomes

We evaluated the effect of BMI on the rate of surgeries for APFT across two BMI categories (30-39 and 20-29) in both unmatched and matched cohorts. In the unmatched analysis, the surgery rate was significantly higher in the BMI 30-39 group (123 surgeries among 969 patients) compared to the BMI 20-29 group (75 surgeries among 790 patients), with an OR of 1.386 (95% CI, 1.023 to 1.878). The mean number of operations was slightly higher in the BMI 20-29 group (1.28 vs. 1.15), but this difference was not statistically significant (p = 0.121).

In the matched cohorts, each containing 733 patients, the surgery rate was significantly higher in the BMI 30-39 group (95 surgeries) compared to the BMI 20-29 group (63 surgeries), with an OR of 1.584 (95% CI, 1.131 to 2.217) (Table 6). The average number of operations was 1.17 and 1.27, respectively, with no significant difference in reoperation rates (p = 0.261).

**Table 6:** Matched incidence of operation and reoperation between BMI groups. ORs and p-values were derived from logistic regression analyses. OR, odds ratio; CI, confidence interval; BMI, body mass index

	No. of patients	No. of patients with surgery	OR (CI)	No. of mean operations	Two operations	Three operations	Four operations	p-value
BMI 30-39	733	95	1.584 (1.131, 2.217)	1.17	9	2	1	0.261
BMI 20-29		63		1.27	11	0	2	

Both analyses indicated a higher surgical incidence in patients with a BMI of 30-39, although the higher mean number of operations in the BMI 20-29 group was not statistically significant, suggesting no increased risk of multiple surgeries between the BMI cohorts.

## Discussion

Management

Initial diagnosis of APFT can be difficult for providers, since this is a clinical diagnosis. Following the literature and assessing for the sensitive physical exam findings is important. After proper diagnosis, management typically hinges on the swift and effective eradication of the infection to prevent spread and further complications, such as tendon damage or systemic infection. The current consensus strongly supports combining surgical intervention and antibiotic therapy as the most effective treatment approach, especially in severe cases. Surgical treatment usually involves incision and drainage of the infected sheath, debridement of necrotic tissue, and sometimes the removal of foreign bodies. This is often followed by systemic antibiotic therapy tailored to culture results or broad-spectrum antibiotics initially [12].

The fact that APFT is a clinical diagnosis complicates rapid and appropriate diagnosis and, therefore, prompt treatment of this condition. The severity of the diagnosis can vary between providers, as this condition is initially diagnosed based on subjective findings. For example, one physician might diagnose APFT with 2/4 Kanavel signs and manage it with IV antibiotics alone; in turn, this patient will likely have a lower chance of mortality due to the nature of a milder infection. Another physician may emergently take a patient to the operating room with 4/4 Kanavel signs and a systemic infection. This patient will ultimately have a higher mortality rate secondary to a more severe infection. Nonetheless, Kanavel signs on physical exam become somewhat of a confounding variable that differs between hospitals and providers. This makes it more difficult to establish an immediate diagnosis and prevent ensuing systemic and morbid infection. 

Implications

The choice between operative and nonoperative treatment options carries significant implications for patient outcomes. Surgical intervention has traditionally been the cornerstone of treatment for APFT, especially in severe cases characterized by abscess formation or when the infection does not respond to antibiotics alone. Surgery aims to quickly reduce the bacterial load and prevent further spread of the infection, thus potentially preventing tendon damage and loss of function [13].

On the other hand, nonoperative treatment, primarily involving IV antibiotics, offers a less invasive approach that has been increasingly considered for cases caught early, before the formation of an abscess. This approach minimizes the risks associated with surgery, such as complications from anesthesia and prolonged recovery times. Recent studies suggest that, with early diagnosis and aggressive antibiotic therapy, nonoperative management can lead to outcomes comparable to surgical interventions in select patient groups [14,15].

Importance of findings

The primary purpose of this study was to characterize national trends in the incidence and prevalence of operative intervention for APFT from 2015 to 2023. Baseline demographic and clinical characteristics were compared between operative and nonoperative cohorts, and secondary analyses evaluated long-term mortality and amputation outcomes.

Because surgical management is preferentially applied to patients with more severe infection, the higher rates of mortality and amputation observed in the operative cohort are best interpreted as markers of disease severity rather than as consequences of surgical treatment. This result can also serve as a statistic while educating patients on the importance of immediate diagnosis and treatment of APFT, to prevent progression of this limb-threatening infection. These findings should be interpreted cautiously, as surgery is typically reserved for patients with more advanced infection, delayed presentation, abscess formation, or failure of antibiotic therapy. Additionally, the marked increase in operative prevalence over the study period may reflect broader changes in coding practices, database capture, clinical recognition, or documentation patterns, rather than a true shift in surgical indications alone. Further investigation using datasets with greater clinical granularity is needed to better define treatment selection and outcomes in APFT.

Limitations

As with a majority of studies analyzing deidentified aggregate data from a large database, researchers are restricted to a limited subset of information on each patient. For example, this presents a barrier to further evaluating the cause or introduction of an infection. With additional information, such as a patient’s history of present illness or physical exam findings, this might provide further insight regarding independent risk factors or the cause of infection.

Another limitation to utilizing large retrospective patient databases is that the data recorded does not allow the researcher to perform granular analyses. For instance, this conglomerate database does not provide further details regarding the severity of infection or a surgeon's threshold to operate versus continuing with IV antibiotics. Specifically, it does not capture the number of Kanavel signs, the presence of an abscess on imaging, or the Michon stage - the variables that most directly inform the decision to operate - and these could not be incorporated into the propensity-score model. Therefore, this information limits the explanatory nature of certain outcomes measured in this study. Despite the limitations of this study, an attempt to control for potential confounders was made. This included propensity score matching of patients by age, gender, race, and ethnicity to ensure the comparison between cohorts was as fair and unbiased as possible, given the observational nature of the data.

## Conclusions

APFT is a serious infection that has demonstrated significant morbidity and, in severe cases, mortality. As the results of this study suggest, several risk factors were associated with a higher likelihood of undergoing surgery, including nicotine dependence, tobacco use, and diabetes. As the incidence and prevalence of APFT continue to rise, this places an even higher priority on educating patients and providers on the clinical features of this limb-threatening condition. The higher mortality and amputation rates observed among operatively managed patients should be understood as reflecting greater infection severity at presentation, rather than an adverse effect of surgery. Another important aspect of this study is that providers should emphasize modifiable risk factors when discussing this condition with patients, to prevent the development of APFT.
